# Immunometabolic reprogramming in the management of prurigo nodularis with methotrexate

**DOI:** 10.1016/j.jdcr.2025.05.038

**Published:** 2025-06-25

**Authors:** Viviane Liao, Junwen Deng, Davies Gage, Anjali D’Amiano, Yagiz Matthew Akiska, Shahin Shahsavari, Madan M. Kwatra, Shawn G. Kwatra

**Affiliations:** a Department of Dermatology, University of Maryland School of Medicine, Baltimore, Maryland; b Maryland Itch Center, University of Maryland School of Medicine, Baltimore, Maryland; c Department of Anesthesiology, Duke University School of Medicine, Durham, North Carolina

**Keywords:** eosinophil, inflammation, methotrexate, plasma cytokine, plasma metabolites, prurigo nodularis

## Introduction

Prurigo nodularis (PN) is a chronic skin disease characterized by inflammatory pruritic nodules distributed primarily on the arms, legs, and trunk. Unfortunately, there are currently limited treatments for PN approved by the Food and Drug Administration, leading to use of off-label therapies like methotrexate.^1^ Methotrexate, a folic acid antagonist, is a common treatment for inflammatory conditions such as rheumatoid arthritis and atopic dermatitis because of its antiproliferative and anti-inflammatory properties. It has also been used in low doses to treat PN and decrease pruritus.^2^ In this case report, we detail a case of refractory PN treated effectively with methotrexate, with novel assessment of accompanying immunometabolic changes.

## Case report

**Fig 1:**
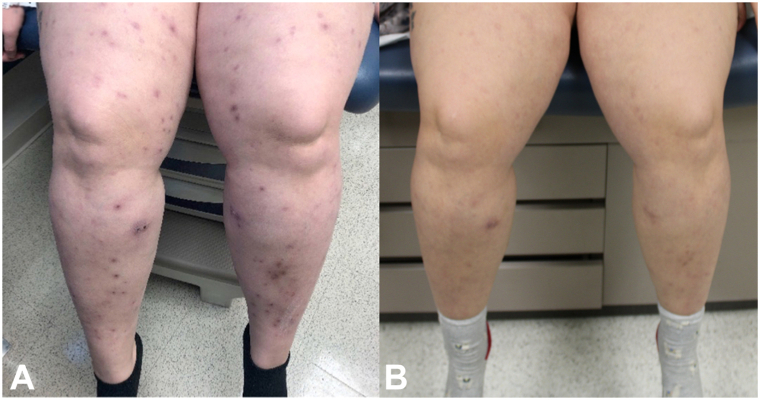
Clinical improvement following methotrexate treatment. Photos of patient’s lower extremities before **(A)** and after **(B)** 8 weeks of treatment with methotrexate, demonstrating marked clinical improvement.

In the present study, a 28-year-old Caucasian woman with a history of Crohn’s disease presented with pruritic, erythematous, nodular papules on her lower and upper extremities, lower abdomen, and back that developed 1 year prior, consistent with PN. She had failed several treatments, including topical corticosteroids, systemic corticosteroids, antihistamines, selective serotonin reuptake inhibitors, dapsone, and UV-B light therapy. Given the refractory nature of her PN the patient was initiated on methotrexate 15 mg weekly, a dosage determined based on the prior literature on methotrexate use in PN and consideration of the patient’s body habitus,^2^ while continuing treatment with triamcinolone 0.1% cream and longstanding UV-B light therapy that had previously elicited limited improvements in pruritus, as well as longstanding adalimumab for Crohn’s disease. Following 8 weeks of methotrexate treatment, the patient had fewer papules on her extremities and trunk and a substantial decrease in her Worst-Itch Numeric Rating Scale from 7/10 to 2/10 (Fig 1, *A, B*). She was titrated up to 20 mg weekly after 3 months and continued on methotrexate for a total of 6 months with excellent response. She was then tapered off and has been maintained on UV-B light therapy with continued clear skin for 2 years following methotrexate treatment.

To further characterize the biologic mechanisms involved in treatment of PN with methotrexate, we measured her eosinophil count, plasma cytokine levels, and plasma metabolite levels before and after clinical improvement. This study was approved by the local Institutional Review Board. Peripheral blood for laboratory tests, including complete blood count, cryopreserved whole blood, and plasma samples were collected immediately before methotrexate treatment and 8 weeks later at the time of clinical improvement. These before- and after-treatment samples were compared to corresponding samples from an age-, sex-, and race-matched healthy control with no pruritic or inflammatory dermatoses. For plasma cytokine analysis, Luminex bead-based immunoassays (Millipore, Burlington, MA) following Immune Monitoring Core standard operating procedures were performed, and the Bioplex 200 platform (Biorad, Hercules, CA) was used to determine the concentration of target plasma proteins. For plasma metabolite analysis, metabolites were extracted from patient plasma, and metabolite data were acquired using a Thermo Scientific Q Exactive Plus Orbitrap Mass Spectrometer with a Vanquish Ultra Performance Liquid Chromatography system (Thermo Fisher, Waltham, MA). Fold changes (FCs) in cytokine and metabolite concentrations were calculated between before- vs after-treatment samples and compared to samples from the healthy control. Metabolite pathways analysis was then performed using Metaboanalyst 5.0, with input parameters comprising the metabolites with FC < 0.5 or > 1.5 post-treatment compared to pretreatment.

**Fig 2:**
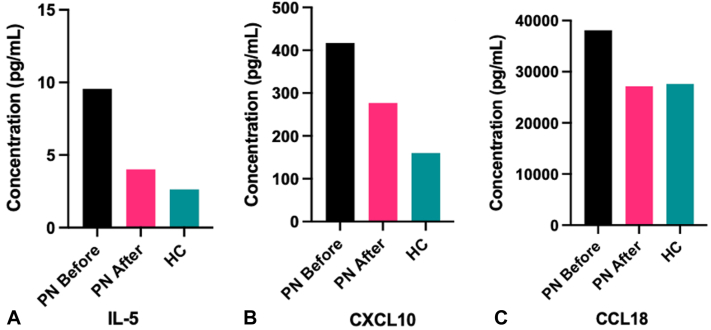
Plasma cytokine changes associated with methotrexate treatment. IL-5 **(A),** CXCL10 **(B),** and CCL18 **(C)** concentrations in blood plasma decreased following methotrexate treatment to levels similar to those found in the healthy control. *HC*, Healthy control; *PN*, prurigo nodularis.

Complete blood count demonstrated the presence of eosinophilia before treatment, which resolved afterwards. The percent eosinophils decreased from 5.5% to 1.6% (normal range 1.0% to 4.0%), and the absolute eosinophil count decreased from 0.33 to 0.09 × 10^3^/μL (normal range 0.12-0.30 × 10^3^/μL). Analysis of plasma cytokines (Fig 2, *A-C*) demonstrated decreases in IL-5 (FC 0.42), CXCL10 (FC 0.66), and CCL18 (FC 0.71) following treatment, with normalization to levels near that of the healthy control. There were no substantial alterations in IL-25 (FC 1.07), IL-31 (FC 1.04), or IL-33 (FC 0.97) cytokine levels.

**Fig 3:**
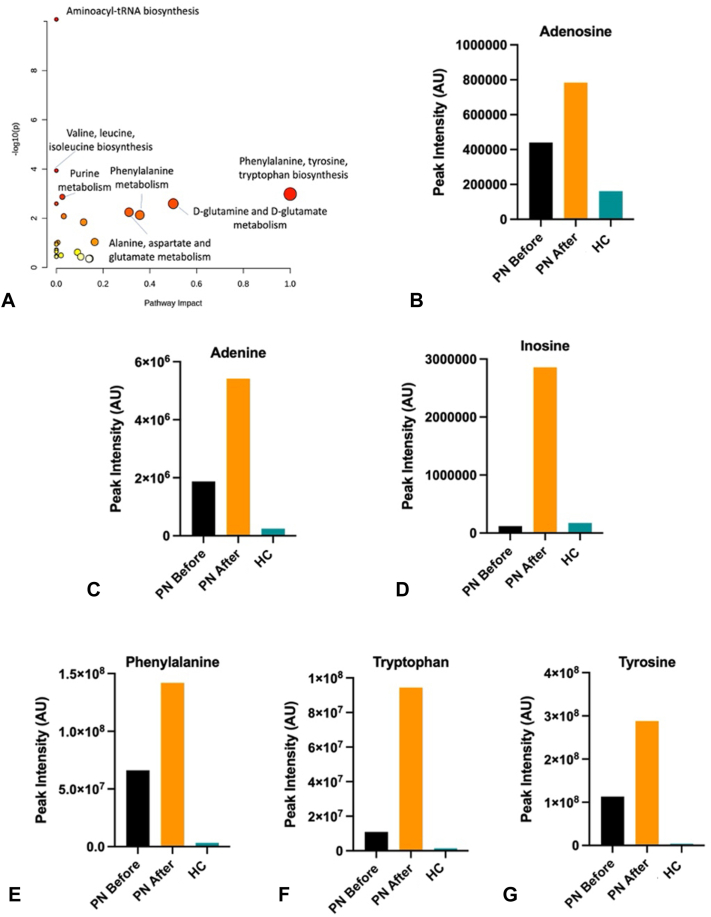
Plasma metabolite changes associated with methotrexate treatment. **A,** Pathway analysis based on changes in concentration of metabolites in blood plasma. The most affected pathways are plotted based on pathway impact and -log10 of the *P* value. Purine pathway metabolites, including adenosine **(B),** adenine **(C),** and inosine **(D)** exceeded healthy control levels following methotrexate treatment. Biopterin pathway intermediates, including phenylalanine **(E),** tryptophan **(F),** and tyrosine **(G),** exceeded healthy control levels following methotrexate treatment. *HC*, Healthy control; *PN*, prurigo nodularis.

Analysis of plasma metabolites (Fig 3, *A*) demonstrated expected increases in purine and biopterin pathway metabolites following methotrexate exposure. We identified 1.78-, 2.88-, and 23.53-fold increases in adenosine, adenine, and inosine (Fig 3, *B-D*), respectively, and 2.15-, 8.59-, and 2.55-fold increases in phenylalanine, tryptophan, and tyrosine (Fig 3, *E-G*), respectively, after treatment. Post-treatment levels of these metabolites exceeded levels found in the healthy control.

## Discussion

We demonstrate that clinical improvement in PN elicited by methotrexate in 1 patient was reflected in profiling of immune cell populations, cytokines, and metabolites in the blood. Low-dose methotrexate has previously been shown to induce eosinophil apoptosis,^3^ as reflected in the reduction of eosinophils after treatment. We corroborate these findings by demonstrating reductions in CXCL10 and CCL18, chemoattractants released by eosinophils, and suggest that methotrexate may also decrease eosinophil activation through reduction of .interleukin (IL) 5.^4^, ^5^, ^6^ Lack of change in IL-25, IL-31, and IL-33 may suggest methotrexate’s effects are mediated more specifically through decrease of eosinophils and related activating cytokines, rather than broader T helper 2 cell (Th2) pathways classically associated with itch. Other mechanisms involved may be increases in the anti-inflammatory effects of adenosine and its associated purine pathway metabolites, adenine and inosine.^7^ Post-treatment increases in phenylalanine, tryptophan, and tyrosine—key precursors for dopamine and serotonin—are consistent with recent findings demonstrating significant dysregulation in this metabolic pathway among PN patients, with slight decreases in tryptophan and tyrosine compared to healthy controls.^8^ Marked upregulation of these metabolites following treatment may reflect changes in itch-related neurotransmission and sympathetic nervous system activation.^8^^,^^9^

Although we analyzed only 1 case, this provides initial insights that inhibition of eosinophil activation and migration, as well as alterations in purine and neurotransmitter metabolism, may be molecular mechanisms involved in the therapeutic response to methotrexate. Further studies should examine a broader population of PN patients to fully characterize how methotrexate affects immunometabolic reprogramming.

## Conflicts of interest

Dr Kwatra is an advisory board member/consultant for Abbvie, Amgen, Arcutis Biotherapeutics, Aslan Pharmaceuticals, Cara Therapeutics, Castle Biosciences, Celldex Therapeutics, Galderma, Genzada Pharmaceuticals, Incyte Corporation, Johnson & Johnson, Leo Pharma, Novartis Pharmaceuticals Corporation, Pfizer, Regeneron Pharmaceuticals, and Sanofi and has served as an investigator for Galderma, Incyte, Pfizer, and Sanofi. The other authors have no conflicts of interest to declare.
